# Timing is critical: consequences of asynchronous migration for the performance and destination of a long-distance migrant

**DOI:** 10.1186/s40462-022-00328-3

**Published:** 2022-06-20

**Authors:** Marta Acácio, Inês Catry, Andrea Soriano-Redondo, João Paulo Silva, Philip W. Atkinson, Aldina M. A. Franco

**Affiliations:** 1 School of Environmental Sciences, University of East Anglia, Norwich, Norfolk UK; 2 CIBIO, Centro de Investigação em Biodiversidade e Recursos Genéticos, InBIO Laboratório Associado, Campus de Vairão, Universidade Do Porto, 4485-661 Vairão, Portugal; 3 CIBIO, Centro de Investigação em Biodiversidade e Recursos Genéticos, InBIO Laboratório Associado, Instituto Superior de Agronomia, Universidade de Lisboa, 1349-017 Lisbon, Portugal; 4 BIOPOLIS Program in Genomics, Biodiversity and Land Planning, CIBIO, Campus de Vairão, 4485-661 Vairão, Portugal; 5 Helsinki Lab of Interdisciplinary Conservation Science (HELICS), Department of Geosciences and Geography, University of Helsinki, Helsinki, Finland; 6 Helsinki Institute of Sustainability Science (HELSUS), University of Helsinki, Helsinki, Finland; 7 British Trust for Ornithology, The Nunnery, Thetford, IP24 2PU UK

**Keywords:** Bird migration, Energy expenditure, GPS tracking, Migration phenology, ODBA, Timing of migration, Weather, White storks

## Abstract

**Background:**

Migration phenology is shifting for many long-distance migrants due to global climate change, however the timing and duration of migration may influence the environmental conditions individuals encounter, with potential fitness consequences. Species with asynchronous migrations, i.e., with variability in migration timing, provide an excellent opportunity to investigate how of the conditions individuals experience during migration can vary and affect the migratory performance, route, and destination of migrants.

**Methods:**

Here, we use GPS tracking and accelerometer data to examine if timing of autumn migration influences the migratory performance (duration, distance, route straightness, energy expenditure) and migration destinations of a long-distance, asynchronous, migrant, the white stork (*Ciconia ciconia*). We also compare the weather conditions (wind speed, wind direction, and boundary layer height) encountered on migration and examine the influence of wind direction on storks’ flight directions.

**Results:**

From 2016 to 2020, we tracked 172 white storks and obtained 75 complete migrations from the breeding grounds in Europe to the sub-Saharan wintering areas. Autumn migration season spanned over a 3-month period (July–October) and arrival destinations covered a broad area of the Sahel, 2450 km apart, from Senegal to Niger. We found that timing of migration influenced both the performance and conditions individuals experienced: later storks spent fewer days on migration, adopted shorter and more direct routes in the Sahara Desert and consumed more energy when flying, as they were exposed to less supportive weather conditions. In the Desert, storks’ flight directions were significantly influenced by wind direction, with later individuals facing stronger easterly winds (i.e., winds blowing to the west), hence being more likely to end their migration in western areas of the Sahel region. Contrastingly, early storks encountered more supportive weather conditions, spent less energy on migration and were exposed to westerly winds, thus being more likely to end migration in eastern Sahel.

**Conclusions:**

Our results show that the timing of migration influences the environmental conditions individuals face, the energetic costs of migration, and the wintering destinations, where birds may be exposed to different environmental conditions and distinct threats. These findings highlight that on-going changes in migration phenology, due to environmental change, may have critical fitness consequences for long-distance soaring migrants.

**Supplementary Information:**

The online version contains supplementary material available at 10.1186/s40462-022-00328-3.

## Introduction

Every year, billions of birds travel from their breeding grounds to the wintering areas and the phenology and routes of those migrants have been shaped to take advantage of predictable weather events [1, 2]. For instance, birds facing long trans-oceanic flights wait for greatest wind assistance before starting their migratory endeavour [3], whereas birds crossing large land barriers, such as the Sahara Desert, may adopt different routes in autumn and spring migration to exploit seasonal tailwinds [4].

In species with asynchronous migrations, in which individuals of a population migrate at significantly different times, the timing and duration of those movements strongly determine the conditions birds experience during migration (e.g., social cues and weather conditions) [5] which can affect their fitness and survival. Many studies have investigated the consequences of changes in the timing of spring migration, generally concluding that spring arrival dates are advancing [6, 7] and that birds arriving earlier at the nesting grounds have higher breeding success [8, 9]. However, earlier spring migrants may experience worse weather conditions during migration, increasing their energy expenditure [10]. The consequences of variability in timing of the autumn migration are less well understood [11, 12] and may be species specific [13, 14], thus more challenging to determine. Nevertheless, autumn migration is marked by high mortality of juveniles on their first migration [15], as well as by the choice of wintering location [16] that may have carry over effects to the remaining annual cycle [17].

Selecting the timing and route that maximizes exposure to favourable weather conditions is particularly important for soaring birds, which rely on supporting winds and strong thermal updrafts to fly [18]. These allow them to soar more efficiently [19] and to minimise energy expenditure [20–22]. Simultaneously, many bird species are highly vulnerable to weather conditions, which can be extremely dynamic and change throughout the migratory season [2]. Weather can enhance bird’s migratory performance by increasing daily speeds and displacements [18, 23], but it can also hinder migratory progression [24], forcing birds to interrupt migration and perform stopovers, while waiting for more favourable weather conditions [25], or lead to mortality [26]. Wind conditions are highly influential, shaping the migratory routes by promoting unintended detours [4] and even determining bird’s wintering areas [16], ultimately influencing population migratory connectivity [27]. Using high spatial and temporal resolution tracking data of long-distance migrants can thus help unravel the effects of exposure to variable weather conditions due to variability in the timing of migration.

In this study, we use GPS tracking data to examine if timing of autumn migration influences the migratory performance and destination, as well as the weather conditions birds experience *en route*, and how these influence bird’s flight directions. Our study focuses on white storks (*Ciconia ciconia*) from a partial migratory population, where some individuals remain in the breeding area all year round, while others still undertake a long-distance migration to the original Sub-Saharan wintering grounds [28]. White storks are large soaring birds exhibiting high variability in the timing of migration [29]. Moreover, the range of the migration period has been increasing over the last few decades [14]. This species has been observed crossing the Strait of Gibraltar all months of the year except in June [29, 30], thus making it an ideal study species to investigate the influence of timing of migration on individual performance.

We use a 5-year GPS tracking dataset with tri-axial acceleration, enabling us to identify bird behaviour and energy expenditure, to understand the consequences of variability in the timing of migration. Specifically, our goal is to examine if timing of migration influences (i) migratory performance i.e., migration duration, distance travelled, route straightness and flight energy expenditure, and (ii) the autumn migration destination in the Sahel. Finally, we examine storks’ exposure to the weather conditions (wind speed, wind direction, and boundary layer height) during migration, and assess the influence of wind direction on bird’s flight direction. This work can provide a mechanistic understanding of the influence of timing of migration on migratory performance and wintering site selection, with potential consequences for migratory connectivity and exposure to anthropogenic threats.

## Methods

### Tracking data and behavioural classification

Between 2016 and 2020, we GPS-tracked 100 first-year juveniles and 72 adult white storks. Among the 72 tracked adults, 10 were long-distance migrants (14%) while the remaining 62 were either residents in Iberia or short-distance migrants to Morocco. This study included 16 adult bird/years (5 adults tracked for 1 migration, 4 adults tracked for 2 migrations, and 1 adult tracked for 3 migrations), and 59 first-year juveniles with completed sub-Saharan autumn migrations, from southern Portugal to the Sahel wintering areas. Adults were trapped at landfill sites, using nylon leg nooses, and at their nests with a remotely activated clap trap. We confirmed that the adults captured on the landfills were of breeding age by identifying their nests using the GPS data, and by visiting the nests to confirm the presence of eggs and/or chicks. Pre-fledging juveniles were retrieved from their nests for tag deployment and returned afterwards. Storks were equipped with GPS/GSM loggers with tri-axial acceleration (“Flyway-50” from Movetech Telemetry, with 4 different models, and “Ornitrack-50” loggers from Ornitela), weighing between 50 and 80 g. The devices were attached as backpacks using a Teflon ribbon thoracic full harness, in total weighing between 1.1 and 2.9% of the bird’s body mass (more details on tag deployment and harnesses in [28, 31, 32]). This study was carried out in agreement with the recommendations of Instituto da Conservação da Natureza e das Florestas and the Animal Welfare & Ethical Review Board from the School of Biological Sciences at the University of East Anglia. Licenses to deploy the loggers were granted by the Instituto da Conservação da Natureza e das Florestas.

The loggers provided 9 consecutive GPS and acceleration fixes at 1 Hz every 20 min during daylight, thus acceleration and location matched in space and time. These acceleration bursts allowed the calculation of Overall Dynamic Body Acceleration (ODBA) and bird behaviour. ODBA is a valid proxy for energy expenditure [33] and it was calculated by subtracting each acceleration point from a running-mean of 4 s for each axis and summing the resulting values for all three axes. To infer bird behaviour, we trained a random forest machine-learning algorithm using 1,000 manually labelled acceleration bursts of 4 different behaviours: foraging, resting, soaring and flapping flight (see Soriano-Redondo et al. [32] for a full description of ODBA and behaviour classifications). To account for disparities in accelerometery sensors of different logger manufacturers, we built separate random forest models for Movetech Telemetry and Ornitela loggers, with 96% and 97% accuracies, respectively. In this study, we combined soaring and flapping flight behaviours into one single *flight* category. If acceleration information was not available, we classified a GPS fix as *flight* if the GPS recorded ground speed was over 5 km/h (15% of total flight classifications).

### Influence of timing on white stork migration performance and destination

**Fig. 1:**
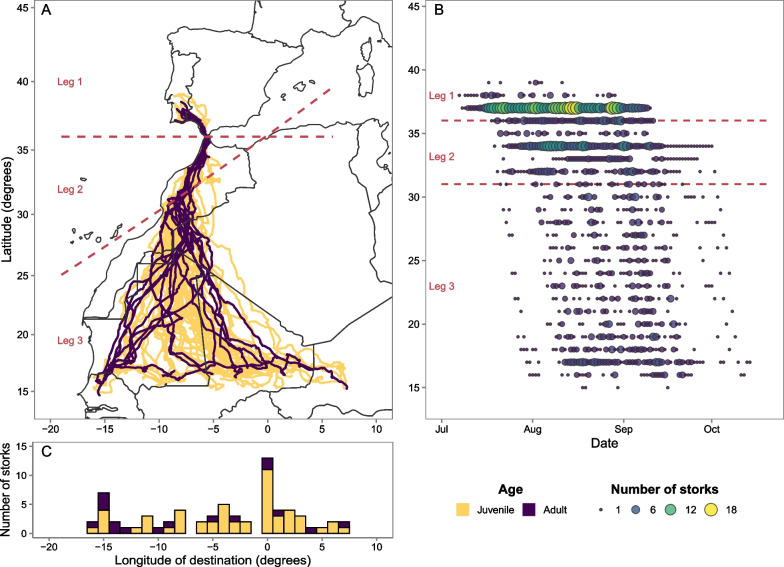
Migration timing, routes, and destination of white storks. **A** Autumn migration routes of adult (purple, n = 16) and juvenile (yellow, n = 59) white storks tracked between 2016 and 2020. Dashed lines indicate the migratory legs: Leg 1—from Portugal until the Strait of Gibraltar; Leg 2—from the Strait of Gibraltar until the Atlas Mountains; Leg 3—from the Atlas Mountains until south of the Sahara Desert. Map was plotted using Mercator projection. **B** Temporal pattern of latitudinal progression of white storks during Autumn migration. Larger and lighter circles indicate a greater number of storks. Leg 2 was represented as 32° latitude, as over 50% of the GPS tracked white storks crossed the Atlas Mountains at that latitude. Latitude was plotted using Cartesian coordinates. **C** Longitude of autumn migration destination, plotted using Cartesian coordinates

White storks travelling to the sub-Saharan wintering locations must cross three major ecological barriers, the Strait of Gibraltar, the Atlas Mountains, and the Sahara Desert. This species can take long stopovers during migration, hence the timing of crossing each ecological barrier is not necessarily correlated for all individuals, as early birds may perform long stopovers and end migration later. Therefore, we divided migration into three legs corresponding to the crossing of ecological barriers (Fig. 1A) and classified the timing of migration as the date storks start each leg: *Leg 1*, from the start of migration in Portugal until the day the stork crosses the Strait of Gibraltar (defined as 36° latitude); *Leg* 2, from the day after crossing the Strait of Gibraltar until the day the stork crosses the Atlas Mountains (defined by the line equation y = 0.58x + 36.12, more details on Additional file 1: S1); *Leg 3*, from the day after crossing the Atlas Mountains until the end of migration, south of the Sahara Desert.

The start and end of autumn migration (i.e., start of leg 1 and end of leg 3) was classified using the spatio-temporal displacement method described in Soriano-Redondo et al. [28]. This method combines movement displacement and spatial and temporal thresholds to define the start and end of migratory movements. Thus, we defined the start of migration as the first of three consecutive days a stork moved more than 60 km between roosts after having left the breeding area (calculated as the 90% kernel of June GPS locations), and the start of wintering period as the first of three consecutive days a stork displaced less than 60 km between roosts after having arrived at the wintering area (calculated as the 90% kernel of October GPS locations). The end of autumn migration was defined as the last day before the start of the wintering period, and the destination of the autumn migration was the location where birds ended autumn migration. All analysis were performed using R package *geosphere* [34] for distance calculations and *adehabitatHR* for kernel estimations [35].

We assessed migratory performance of individual white storks using several metrics: migration duration (in days), total number of migratory and stopover days, total migration distance (in kms), straightness of the migratory route (index between 0–1) and mean flight energy expenditure (in G). These metrics were calculated for the overall migration and for each leg, and we only included individuals for which we had daily data for the entire period.

Migration duration and leg duration were determined as the difference in days between the start and end of migration, and between the start and end of each migration leg. Stopover and migratory days corresponded to days when birds moved less or more than 37 km between consecutive night roosts, respectively (see Additional file 1: Appendix S2 for a description of the calculation of stopover and migratory days’ threshold).

Total migration distance (hereafter, beeline distance) is the shortest great-circle (i.e., orthodromic) route between the start and end locations of autumn migration. White storks from the Portuguese population must cross the Strait of Gibraltar on their way to Africa, hence we calculated the migration beeline distance as the distance between the first autumn migration GPS location and the location at the Strait of Gibraltar and summed it to the migration distance from the Strait of Gibraltar to the arrival location in the Sahel. The beeline distance of each migratory leg was calculated as the shortest orthodromic route between the GPS location at the start of the leg and the first GPS position of the subsequent leg, or, for leg 3, the last GPS position of autumn migration.

We calculated the cumulative travelled distance as the sum of the distances between all daylight GPS fixes on migratory days. Route straightness was defined as the ratio between the beeline distance and the cumulative distance travelled [4], for the whole migration and for each migratory leg. Finally, we summarised white storks’ flight energy expenditure as the mean ODBA of all accelerometer bursts classified as *flight*.

The influence of timing of migration on migratory performance was examined using separate models for each performance metric and migratory leg, to account for differences in geography and weather of the different legs, while minimizing the complexity of the models. In total, we built 18 models, for 6 migration metrics on the 3 migratory legs. Using the R package *lme4* [36], we built linear mixed models (LMMs), with gaussian distribution and identity link function, with migration duration, number of stopover and migratory days, beeline distance and mean flight energy expenditure as response variables, with timing of start of leg (in Julian day) and age as fixed effects (to account for possible age differences in performance), and bird ID and year as random effects. For mean flight energy expenditure, we also added logger type as a random effect, to control for differences in ODBA estimates between loggers with different accelerometer sensors. To analyse the influence of timing of migration on route straightness, we built a generalised linear mixed model (GLMM), with beta distribution and logit link function, using *glmmTMB* package [37], with route straightness as the response variable, and timing of start of leg and age as fixed effects, and bird ID and year as random factors.

We built two LMMs, with gaussian distribution and identity link function, with latitude and longitude of the destination of autumn migration as response variables to examine the influence of timing on the location where birds finish autumn migration. Timing of start of leg (in Julian day) and age were included as fixed effects, and bird ID and year as random effects. For all LMMs we estimated *p-values* using *car* package [38], and pseudo-R^2^ using *MuMIn* package [39], and multicollinearity was analysed by verifying the variance inflation factor (VIF < 2 [40]).

#### Influence of timing on the weather conditions experienced by white storks

To examine the weather conditions individual birds experienced, we firstly annotated the white stork GPS locations on migratory days with hourly weather data from ERA-5 [41, 42], with 30 km spatial resolution, using the *GAMT* R package (Bird et al., unpublished data) and bilinear interpolation. Similarly to other studies of migratory soaring birds [18, 43, 44], we selected boundary layer height (a proxy for the depth of the vertical columns of rising air, commonly named thermals, from which soaring birds profit to gain altitude during flight [18]) and wind zonal (i.e. westward(-)/eastward(+)) and meridional (i.e. southward(-)/northward (+)) components at 925 mB (corresponding to approximately 700 m, which is the mean flight altitude for soaring birds on migration [16]). Using wind zonal and meridional components, we calculated for each GPS location the wind speed and direction, as well as the wind support each bird experienced [45, 46].

We summarised the weather conditions experienced by white storks on each leg as the mean boundary layer height, mean wind support, and mean zonal wind speed. The mean value was used as it summarises the average conditions birds experienced, while also considering the extreme weather conditions storks might have encountered during migration. We then fitted LMMs, with gaussian distribution and identity link function, with the summarised weather variables as response variables, with timing of start of leg (in Julian day) and age as fixed effects, and bird ID and year as random effects.

#### Influence of timing and wind direction on white stork flight direction

The crossing of the Sahara Desert (leg 3) is a critical stage of migration, as the high temperatures and the almost complete absence of food and water [47] make it a mortality hotspot for many bird species [48]. This is also the final stage of migration before white storks reach the sub-Saharan wintering grounds, so any deviations to the migration route may determine where storks finish migration. To determine if the timing of crossing leg 3 and the direction of the winds influence stork’s flight direction, we examined the daily movements of white storks during the crossing of the Sahara Desert in more detail. First, we determined bird’s ground speed and bearing on GPS *flight* fixes, by calculating the time, distance, and direction between two consecutive locations. We then derived stork’s flight direction as the longitudinal speed (i.e. westward(−)/eastward(+)) at each GPS location [16] and calculated the mean daily longitudinal speed. Using the weather data, we calculated the mean daily zonal wind speed experienced by the storks when flying. Finally, using a LMM, with gaussian distribution and identity link function, we assessed if the start date of leg 3, daily mean zonal wind speed, and their interaction affected the mean stork longitudinal speed. Bird ID and year were included as random effects.

## Results

Between 2016 and 2020 we GPS-tracked 172 white storks and recorded 75 complete sub-Saharan autumn migration tracks (adults = 16, juveniles = 59), consisting of 1235 migratory days and 900 stopover days. In total, the dataset comprised 96,630 GPS fixes (adults = 17,638, juveniles = 78,992), with an average of 1288 GPS fixes per track (sd = 809). Storks began their autumn migration over a two-month period, between the 7th of July and 4th of September (median date = 5th of August, sd = 17 days) and arrived at the wintering grounds between the 31st of July and 14th of October (median date = 6th of September, sd = 15 days), travelling more than 2500 km (adults = 2563 ± 38.4 km, juveniles = 2525 ± 15.3 km), and taking on average 25 (adults, sd = 12) and 31 (juveniles, sd = 17) days (Table 1). Both age groups finished their migration over a large area in the Sahel region, spanning 2450 km, from Senegal to Niger (Fig. 1).

**Table 1:** Summary of adult and juvenile white stork migration characteristics for the whole migration and for the three different migration legs: Leg 1—from Portugal until the Strait of Gibraltar; Leg 2—from the Strait of Gibraltar until the Atlas Mountains; Leg 3—from the Atlas Mountains until south of the Sahara Desert

Migratory Leg	Age	Mean start date (range)	Mean migration duration, in days (SE)	Mean number of migration days (SE)	Mean number of stopover days (SE)	Mean beeline distance, in km (SE)	Mean route straightness index (SE)	Mean latitude of destination, in degrees (range)	Mean longitude of destination, in degrees (range)
All migration	Adult	–	25 (3.1)	16 (0.5)	10 (3.0)	2563 (38.4)	0.73 (0.02)	16.68 (14.65–17.94)	− 7.28 (− 15.51 to 7.27)
	Juvenile	–	31 (2.2)	18 (0.3)	14 (2.1)	2525 (15.3)	0.67 (0.01)	16.67 (15.25–17.98)	− 3.24 (− 15.53 to 7.03)
Leg 1	Adult	10.Aug (09.Jul–03.Sep)	9 (2.2)	4 (0.3)	6 (2.1)	376 (11.2)	0.71 (0.03)	–	–
	Juvenile	04. Aug (07.Jul–04.Sep)	10 (1.4)	4 (0.1)	6 (1.4)	374 (7.6)	0.63 (0.01)	–	–
Leg 2	Adult	20. Aug (21.Jul–12.Sep)	6 (1.4)	3 (0.1)	3 (1.4)	562 (21.0)	0.85 (0.02)	–	–
	Juvenile	14.Aug (19.Jul–10. Sep)	9 (1.5)	3 (0.2)	6 (1.4)	504 (17.2)	0.78 (0.02)	–	–
Leg 3	Adult	26.Aug (06.Aug–15.Sep)	11 (1.1)	9 (0.6)	2 (0.6)	1679 (53.0)	0.74 (0.02)	–	–
	Juvenile	24.Aug (22.Jul–03.Oct)	12 (0.4)	11 (0.3)	1 (0.3)	1723 (21.9)	0.69 (0.01)	–	–

### Influence of timing on white stork migratory performance and arrival at the wintering grounds

**Fig. 2:**
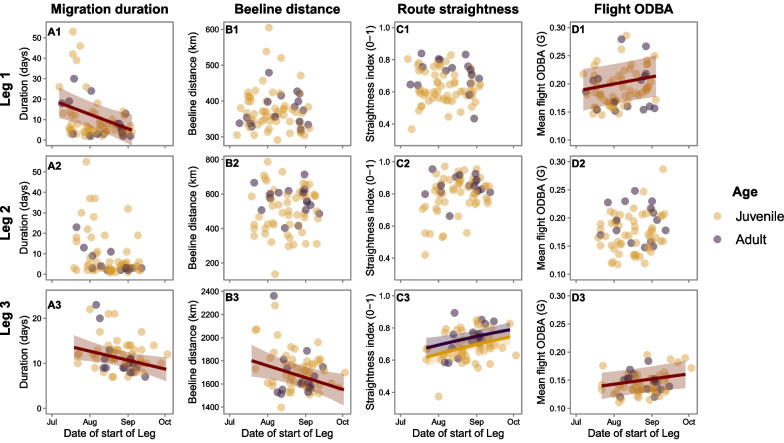
Influence of timing of starting leg 1, leg 2 and leg 3 on white stork migration performance. Model predictions of the relationship between the starting date of migratory legs and **A1–A3** migration duration (days), **B1–B3** beeline migratory distance (km), **C1–C3** route straightness (0–1), and **D1–D3** mean flight energy expenditure (ODBA, G). Data was modelled using linear and generalised linear mixed models, with gaussian (models A, B and D) and beta distributions (models C), using bird id and year, and (models D) logger model, as random factors. Red lines show the statistically significant relationships, and purple and yellow lines highlight the statistically significant differences between adult and juvenile performance, respectively. Shading represents 95% confidence intervals and points show the raw data for adults (purple) and juveniles (yellow)

Timing of migration significantly influenced migratory performance for all metrics analysed, but at different stages of the migration (Fig. 2, statistic in Tables 2, 3). Storks starting leg 1 and leg 3 earlier took longer to complete the respective legs, spending more days on stopovers on leg 1, and adopting longer and less direct routes on leg 3. Early storks also spent less energy when flying on leg 1 and leg 3. When comparing the migratory performance of adults and juveniles, we found that adults adopted significantly straighter routes than juveniles when crossing leg 1 and leg 3, but found no differences in flight energy expenditure.

**Table 2:** Influence of timing on migration duration and number of stopover and migratory days

Response	Leg	Predictor	Estimate (SE)	t value	*p* value	R^2^ marginal	R^2^ conditional
Migration duration	1	Intercept	11.51 (3.15)	3.66	–	0.12	0.88
		Date	− 3.92 (1.24)	− 3.17	0.002**		
		Age (juv.)	− 1.86 (3.38)	− 0.55	0.582		
	2	Intercept	7.26 (3.44)	2.11	–	0.01	0.88
		Date	0.07 (0.55)	0.14	0.893		
		Age (juv.)	1.79 (3.73)	0.48	0.632		
	3	Intercept	11.10 (0.88)	12.68	–	0.09	0.09
		Date	− 1.10 (0.47)	− 2.34	0.019*		
		Age (juv.)	0.78 (1.00)	0.77	0.439		
Migratory days	1	Intercept	3.73 (0.31)	11.95	–	0.01	0.12
		Date	− 0.07 (0.13)	− 0.58	0.565		
		Age (juv.)	0.19 (0.31)	0.62	0.534		
	2	Intercept	2.69 (0.41)	6.54	–	0.05	0.75
		Date	− 0.11 (0.15)	− 0.70	0.483		
		Age (juv.)	0.62 (0.41)	1.49	0.136		
	3	Intercept	9.40 (0.56)	16.88	–	0.13	0.13
		Date	− 0.78 (0.30)	− 2.61	0.009**		
		Age (juv.)	1.10 (0.64)	1.72	0.085		
Stopover days	1	Intercept	8.08 (3.10)	2.61	–	0.11	0.95
		Date	− 3.65 (1.18)	− 3.09	0.002**		
		Age (juv.)	− 2.39 (3.35)	− 0.71	0.475		
	2	Intercept	1.70 (0.51)	3.32	–	0.02	0.02
		Date	− 0.32 (0.27)	− 1.16	0.244		
		Age (juv.)	− 0.32 (0.59)	− 0.55	0.581		
	3	Intercept	1.87 (0.62)	3.02	–	0.03	0.62
		Date	− 0.29 (0.28)	− 1.04	0.296		
		Age (juv.)	− 0.49 (0.68)	− 0.72	0.472		

Results of the LMMs, testing the influence of timing of white stork migration and age on migration duration and on the number of migratory and stopover days, using bird ID and year as random factors. The variable “Date” has been scaled by subtracting the mean date and dividing by the standard deviation

**Table 3:** Influence of timing of migration on white stork migratory performance

Response	Leg	Predictor	Estimate (SE)	t/z value	*p* value	R^2^ marginal	R^2^ conditional
Beeline distance	1	Intercept	449.4 (88.0)	5.11	–	0.01	0.80
		Date	− 0.32 (0.39)	− 0.82	0.412		
		Age (juv.)	− 6.59 (18.5)	− 0.36	0.722		
	2	Intercept	499.3 (230.5)	2.17	−	0.04	0.59
		Date	0.27 (0.99)	0.27	0.787		
		Age (juv.)	− 55.92 (41.07)	− 1.36	0.173		
	3	Intercept	2470.4 (328.2)	7.53	–	0.10	0.23
		Date	− 3.35 (1.37)	− 2.45	0.014*		
		Age (juv.)	47.00 (51.84)	0.91	0.365		
Route straightness	1	Intercept	0.91 (0.12)	7.89	< 0.001***	–	–
		Date	− 0.06 (0.05)	− 1.10	0.270		
		Age (juv.)	− 0.39 (0.13)	− 3.06	0.002**		
	2	Intercept	1.79 (0.25)	7.24	< 0.001****	–	–
		Date	0.18 (0.10)	1.77	0.077		
		Age (juv.)	− 0.40 (0.26)	− 1.58	0.115		
	3	Intercept	1.03 (0.10)	9.90	< 0.001***	−	–
		Date	0.15 (0.05)	2.70	0.007**		
		Age (juv.)	− 0.25 (0.12)	− 2.13	0.034*		
Flight ODBA	1	Intercept	0.202 (0.018)	11.32	−	0.04	0.59
		Date	0.007 (0.003)	2.08	0.038*		
		Age (juv.)	0.014 (0.008)	1.62	0.104		
	2	Intercept	0.200 (0.019)	10.30	–	0.01	0.81
		Date	0.003 (0.004)	0.96	0.340		
		Age (juv.)	− 0.005 (0.010)	− 0.53	0.594		
	3	Intercept	0.150 (0.011)	13.39	–	0.04	0.89
		Date	0.005 (0.002)	2.23	0.026*		
		Age (juv.)	0.002 (0.006)	0.44	0.661		

Results of the LMMs (and GLMM, for route straightness), testing the influence of timing of white stork migration and age on migration beeline distance, route straightness and flight ODBA, using bird ID and year as random factors, and on the flight ODBA model, also adding logger model as a random factor. For the route straightness and flight ODBA models, the variable “Date” has been scaled by subtracting the mean date and dividing by the standard deviation. Pseudo-r squared values are not available for GLMMs with beta distributions

**Fig. 3:**
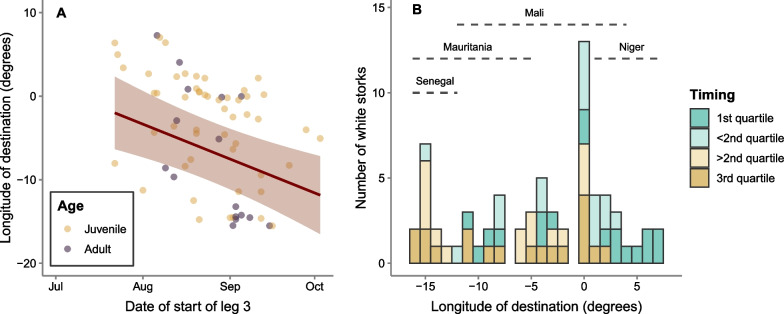
Influence of timing of start leg 3 (crossing the Sahara Desert) on the destination of autumn migration. **A** Model predictions of the relationship between the start date of leg 3 and the longitude of autumn migration. Data was fitted with linear mixed models with gaussian distribution, using bird id and year as random factors. Red lines show the statistically significant relationship, shading represents 95% confidence interval and points show the raw data for adults (purple) and juveniles (yellow). **B** Longitude of the autumn migration destination in the Sahel for early and late migrant white storks. Early birds starting leg 3 on the 1st and 2nd quartile of the migration dates are represented as dark blue and dark brown, respectively, while late birds (later than the median) are represented as light blue and light brown, respectively

The latitude of autumn migration destinations did not differ between early and late migrants (for none of the migratory legs), but longitude was significantly influenced by the timing of migration: storks migrating earlier on all migratory legs were more likely to arrive to Eastern Sahel, while later migrants were more likely to arrive to Western Sahel (Fig. 3). Adults and juveniles finished their migration in similar areas (Table 4).

**Table 4:** Influence of timing on migration destination

Response	Leg	Predictor	Estimate (SE)	t value	*p* value	R^2^ marginal	R^2^ conditional
Latitude of autumn migration destination	**1**	Intercept	17.04 (1.20)	14.17	−	< 0.01	0.08
		Date	0.00 (0.01)	− 0.27	0.791		
		Age (juv.)	− 0.03 (0.22)	− 0.14	0.893		
	**2**	Intercept	17.28 (1.31)	13.14	−	< 0.01	0.08
		Date	0.00 (0.01)	− 0.43	0.668		
		Age (juv.)	− 0.03 (0.22)	− 0.15	0.884		
	**3**	Intercept	17.78 (1.34)	13.29	−	< 0.01	0.09
		Date	0.00 (0.01)	− 0.80	0.423		
		Age (juv.)	− 0.02 (0.21)	− 0.12	0.908		
Longitude of autumn migration destination	**1**	Intercept	12.57 (9.25)	1.36	−	0.11	0.97
		Date	− 0.09 (0.04)	− 2.10	0.036*		
		Age (juv.)	3.25 (2.11)	1.54	0.124		
	**2**	Intercept	25.03 (9.19)	2.73	−	0.18	0.97
		Date	− 0.14 (0.04)	− 3.50	< 0.001***		
		Age (juv.)	3.07 (1.99)	1.55	0.122		
	**3**	Intercept	25.36 (9.20)	2.76	−	0.17	0.97
		Date	− 0.13 (0.04)	− 3.53	< 0.001***		
		Age (juv.)	3.28 (1.99)	1.65	0.099		

Results of LMMs, testing the relationship between the latitude and longitude of the migration destination of white storks, and the date of start each Leg and age of the bird, using bird id and year as random factors

### Influence of timing on the weather conditions experienced by white storks

**Fig. 4:**
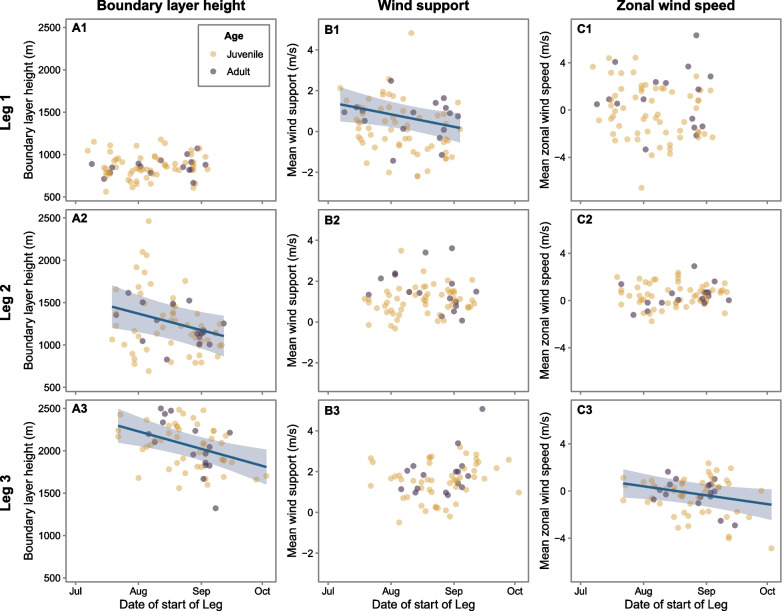
Influence of timing of starting leg 1, leg 2 and leg 3 on the weather conditions experienced by white storks. Model predictions (blue line) of the relationship between white stork timing of migration and **A1–A3** boundary layer height (m) and **B1–B3** mean wind support (m/s), and **C1–C3** mean zonal wind speed (m/s). Data was modelled using linear mixed models, with gaussian distributions, using bird id and year as random factors. Blue lines show the statistically significant relationships, shading represents 95% confidence intervals and points show the raw data for adults (purple) and juveniles (yellow)

**Fig. 5:**
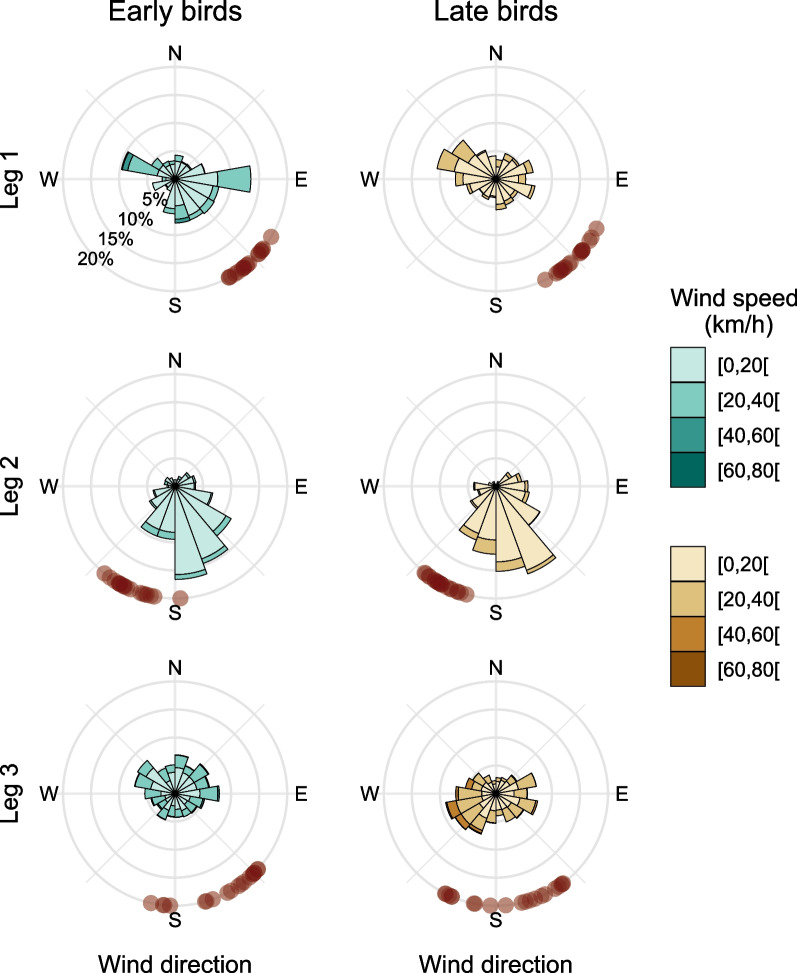
Wind conditions experienced by the birds crossing each migratory leg on the 1st (early birds) and 3rd (late birds) quartile of the migration period. Wind direction and wind speed (both in % of GPS flight fixes) are represented by the length and colour of the bars. Red points show the directions taken by the birds when crossing the migratory leg, calculated as the bearing between the first and last GPS location of the leg

The weather conditions varied along the season (Figs. 4, 5, statistics in Table 5): early birds encountered higher boundary layers (i.e., stronger thermals) on leg 2 and leg 3 and more supportive wind conditions on leg 1 (Fig. 4). Earlier migrants also experienced significantly stronger westerly winds (i.e., winds blowing to the east) on leg 3 (Fig. 5). We only found age related differences in leg 2, juveniles travelled on days with less supportive winds, compared to adults (Table 5).

**Table 5:** Influence of timing of migration on the weather conditions

Response	Leg	Predictor	Estimate (SE)	t value	*p* value	R^2^ marginal	R^2^ conditional
Boundary layer height	1	Intercept	649.8 (208.9)	3.11	−	0.01	0.56
		Date	0.92 (0.93)	1.00	0.379		
		Age (juv.)	3.37 (43.35)	0.08	0.937		
	2	Intercept	2710.2 (612.6)	4.42	−	0.08	0.73
		Date	− 6.29 (2.63)	− 2.39	0.017*		
		Age (juv.)	− 17.64 (111.8)	− 0.16	0.875		
	3	Intercept	3648.9 (507.9)	7.18	−	0.13	0.15
		Date	− 6.67 (2.11)	− 3.16	0.002**		
		Age (juv.)	15.26 (77.52)	0.20	0.844		
Wind support	1	Intercept	5.020 (1.92)	2.61	−	0.09	0.09
		Date	− 0.020 (0.01)	− 2.31	0.021*		
		Age (juv.)	− 0.557 (0.35)	− 1.61	0.107		
	2	Intercept	1.315 (1.37)	0.96	−	0.06	0.15
		Date	0.001 (0.01)	0.18	0.860		
		Age (juv.)	− 0.449 (0.22)	− 2.02	0.043*		
	3	Intercept	− 1.152 (1.71)	− 0.67	−	0.06	0.31
		Date	0.013 (0.01)	1.78	0.074		
		Age (juv.)	− 0.334 (0.28)	− 1.21	0.228		
Zonal wind speed	1	Intercept	5.677 (3.66)	1.55	−	0.05	0.12
		Date	− 0.020 (0.02)	− 1.29	0.197		
		Age (juv.)	− 1.201 (0.67)	− 1.79	0.073		
	2	Intercept	− 0.368 (1.61)	− 0.23	−	< 0.01	0.19
		Date	0.003 (0.01)	0.54	0.590		
		Age (juv.)	− 0.019 (0.26)	− 0.07	0.942		
	3	Intercept	5.673 (2.78)	2.04	–	0.06	0.87
		Date	− 0.025 (0.01)	− 2.13	0.033*		
		Age (juv.)	− 0.207 (0.51)	− 0.40	0.688		

Results of LMMs, testing the relationship between weather conditions white storks experienced (mean boundary layer height, mean wind support, and mean zonal wind speed) and timing of migration and age, with bird ID and year as random effects

### Influence of timing and wind direction on white stork flight direction

**Fig. 6:**
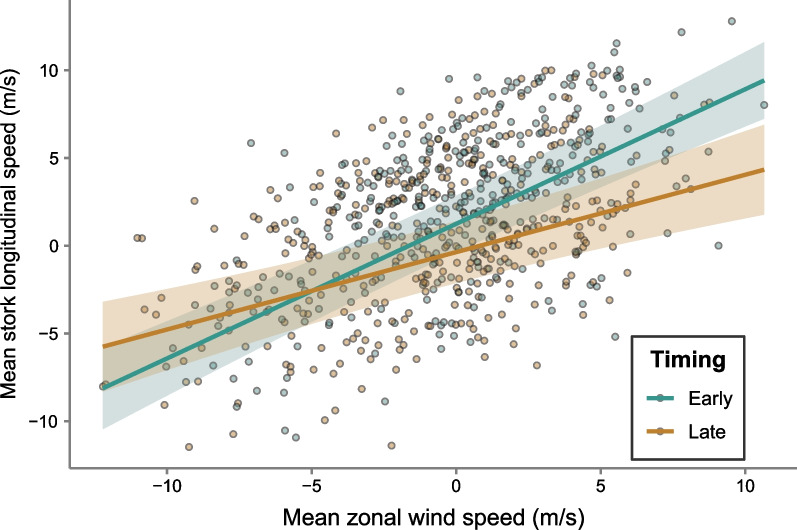
Influence of timing and wind direction on white stork flight direction. Influence of zonal wind speed (m/s) on white stork longitudinal speed (m/s) when crossing migratory leg 3 (from the Atlas Mountains until south of the Sahara Desert), with a significant interaction with timing of start migratory leg 3. Data was modelled using a linear mixed model, with bird id and year as random factors. Blue and brown lines indicate the model prediction for the earliest (22nd July) and latest (3rd October) bird, respectively, and shading represents 95% confidence intervals. Points show the raw data, with individuals starting leg 3 on a date earlier or later than the median date (2nd quartile) coloured as light blue and light brown, respectively

White stork’s longitudinal speed was significantly influenced by the wind’s zonal speed, with significant differences between adults and juveniles in longitudinal speed (statistics in Table 6). A significant interaction between wind zonal speed and timing of migration indicated that earlier migrating storks were more influenced by wind direction than later birds (Fig. 6, Table 6).

**Table 6:** Influence of timing and wind direction on white stork flight direction

Response	Predictor	Estimate (SE)	t value	*p* value	R^2^ marginal	R^2^ conditional
Bird longitudinal speed	(Intercept)	5.740 (3.72)	1.55	–	0.33	0.49
	Date	− 0.022 (0.02)	− 1.42	0.198		
	Age (juv.)	1.506 (0.69)	2.19	0.028*		
	Zonal wind speed	1.674 (0.45)	3.73	< 0.001***		
	Date: zonal wind speed	− 0.004 (0.002)	− 2.36	0.019*		

Results of LMM, testing the relationship between bird longitudinal speed and age, timing of migration, zonal wind speed, and an interaction between these two fixed effects, with bird ID and year as random effects

## Discussion

Successful migration is highly dependent on the weather conditions individuals face [47] and, in asynchronous migratory populations, individuals may experience different environmental conditions throughout their migration, with potential consequences for individual fitness and population connectivity [5]. We studied a soaring bird species, with highly asynchronous migratory movements: counts in the Strait of Gibraltar show that white storks migrating through the western flyway cross the Strait between July and October (median in 11th August) [29], corroborating our results obtained with the Portuguese population. This large variability in the timing of migration determines the environmental conditions individuals encounter *en route* and influences the migratory performance and arrival destination in the Sahel. White storks crossing Iberia and the Sahara Desert earlier in the season encounter more supportive wind and better thermal conditions, spend less energy when flying, and face stronger westerly winds in the Sahara (i.e., winds blowing to the east), leading to increased probability of finishing migration in eastern Sahel, whereas later storks are exposed to winds blowing west and more likely to go to western Sahel.

### Influence of timing on weather conditions and migratory performance

Weather conditions experienced by travelling birds play a critical role in determining flight energy expenditure [49] and migratory performance [18, 23]. Late departing storks were confronted with poorer weather conditions, facing less wind support and weaker thermals, hence spent more energy when flying. Despite the additional energy expenditure in Iberia and in the Sahara, late migrants adopted shorter and straighter routes and spent fewer days on stopovers, possibly as a response to progressively deteriorating weather conditions in autumn. These results suggest that earlier birds stop more often to minimize energy expenditure, whilst later birds tend to minimize migration time [50].

Winds are particularly important in shaping the migratory journeys of soaring birds [2, 43, 44, 51]. We found that the zonal wind direction explained 33% of the variation in white stork longitudinal movements when crossing the Sahara, a lower variance when compared with lone-migrant juvenile honey buzzards (*Pernis apivorus*) [16], suggesting that white stork movements are determined by a combination of environmental variables and other (e.g. social) cues [52]. However, wind conditions are highly dynamic and change throughout the migration season. In the Desert, the easterly winds, known as the ‘Harmattan winds’, are particularly strong towards the end of the migration season (September – November), hence later birds finishing their migration in central Sahel had to challenge the dominant winds to successfully reach the wintering sites.

When crossing the Sahara Desert, adult storks adopted straighter routes compared to juveniles. Birds improve their migratory performance as they age [50] and, since adult white storks are consistent in their migration timing and wintering areas (Additional file 1: Appendix S3), they likely optimise their migration route in consecutive years [53]. Unlike results from other studies [54], we did not find significant differences in flight energy expenditure (i.e., ODBA) between adults and juveniles. Firstly, we only included in the analysis birds that successfully completed the autumn migration. As juveniles with higher energy costs are more likely to perish on migration [54], this could have minimised the differences between age groups. Moreover, it is possible that juveniles from southern populations have more time to improve their flight performance before and during migration, when compared to central-European juveniles: they have more time between fledging and start of migration (median German = 32 days [15], median Portugal = 37 days), and they stop more often while on migration (71% of German storks migrate non-stop [54], compared to 14% of the Portuguese storks). These extra learning periods could thus minimise the differences between adults and juveniles.

### Influence of migration timing on destination

The wintering area of white storks migrating through the western flyway covers a large area of the Sahel [15, 28, 55] and we found that the weather conditions experienced during migration influenced where storks finished their autumn migration in the Sahel: late migrants, exposed to stronger easterly winds, were more likely to end migration in western Sahel, while earlier migrants had a higher probability of ending migration in eastern areas. As expected for this social migratory species, this pattern is true for both adults and juveniles. Adult white storks, as other soaring species, show high repeatability in the timing of migration [56], which possibly exposes storks to similar environmental conditions during migration, directing them to similar wintering areas in successive years (Additional file 1: Appendix S3). Conversely, the population asynchronous migration timing, exposes individuals to different weather conditions, conducting them to distinct areas in the Sahel.

The choice of wintering grounds can influence individual energy expenditure [55, 57] and can also determine annual survival [15, 57, 58]. In fact, severe droughts in the Sahel have been associated with increased over-winter mortality and white stork European population declines in the 70 s [59]. The Sahel region comprises several different habitats [60], which have been subjected to a variety of human-induced changes threatening bird species (from habitat conversion to increased hunting activity [61]), thus individuals wintering in distinct areas might be exposed to different threats [60, 62]. While there is strong evidence that white stork’s spring migratory phenology has been changing, with an advancement of the dates of arrival at the Iberian breeding grounds [63, 64], the spatial and temporal patterns of autumn departure are still unclear [64]: some studies report an advancement on the timing of autumn migration [65], while others describe a delay in the crossing of the Strait of Gibraltar [14] or no significant change in the last decade [66]. An advancement of the autumn migration dates may lead more storks to winter in eastern longitudes, where there could be increased hunting pressure. While storks are hunted throughout their Sahelian wintering areas [67], white storks are known to be persecuted in Niger and Nigeria [68]. Conversely, for white storks delaying their autumn migration, exposure to poor weather conditions *en route* may lead to higher energy expenditure, which can increase mortality during migration [54]. Current climate predictions show that wind strength and storm frequency will increase in the Sahara region [69], but the effects of these changes on migratory birds crossing the Sahara are still unknown [26]. It is therefore important that future studies continue to assess the mortality risks associated with travelling at different times during the migratory season, and to understand the threats birds face in their wintering ranges, to have a full understanding of how on-going autumn phenology and climate change will impact sub-Saharan migrants.

## Conclusions

Using multi-year GPS and acceleration dataset of migrating white storks, we showed the timing of migration influences the weather conditions individuals face, the energetic costs of migration, and the wintering destinations, where birds may be exposed to different environmental conditions and distinct threats (e.g., hunting pressure). These findings highlight that on-going changes in migration phenology, due to environmental change, may have critical fitness consequences for long-distance soaring migrants. We encourage future work to assess the mortality risks associated with different migration timing and wintering destinations, to fully understand how on-going environmental change will affect sub-Saharan migrants.

## Supplementary Information

**Additional file 1**. Description of the definition of stopover days, of the line defining the end of Leg 2 (Atlas Mountains), and of the consistency in timing and destination of adult white storks.

## Data Availability

The tracking data used in this study are stored in Movebank (http://www.movebank.org, study names “White Stork Adults and Juveniles 2016”, “White Stork Adults 2017”, “White Stork Juveniles 2017”, “White Stork Adults 2018”, “White Stork Juveniles 2018”, “White Stork Adults 2019”, “White Stork Juveniles 2019”, “White Stork Adults 2020”, “White Stork Juveniles 2020”) and will be available in the Movebank Data Repository (10.5441/001/1.137cn005) after a one-year embargo, or on request. ERA-5 weather data were downloaded from the Copernicus Climate Change Service (C3S) Climate Data Store (CDS) (10.24381/cds.bd0915c6).
