# Supplementary material for: Timing is critical: consequences of asynchronous migration for the performance and destination of a long-distance migrant
Source: Mov Ecol. 2022 Jun 20;10:28. doi: 10.1186/s40462-022-00328-3 (PMC9901525; doi:10.1186/s40462-022-00328-3)
Supplement: Supplementary file 1 — Additional file 1. Description of the definition of stopover days, of the line defining the end of Leg 2 (Atlas Mountains), and of the consistency in timing and destination of adult white storks. [file 40462_2022_328_MOESM1_ESM.docx]

**Timing is critical: Consequences of asynchronous migration for the performance and destination of a long-distance migrant**

**Supporting Information**

**Appendix S1**

**Line equation defining the end of Leg 2 (corresponding to the Atlas Mountains)**

The Atlas is a mountain range in North Africa extending from Morocco to Algeria. Due to the diagonal range of these mountains, to identify the day when white storks cross this barrier we could not rely on the crossing of a single latitudinal value. Thus, we calculated the line equation that connected two points in Morocco that delimitate the southwest and northeast borders where the tracked white storks crossed the Atlas Mountains: Tamraght (y1=30.505321, x1=-9.652890) and Tassader (y2=33.509944, x2=-4.488463). This way, the end of Leg 2 is the day when white storks cross the line given by the following equation:

y = 0.58x + 36.12,

in which 0.58 is the slope of the line and 36.12 is the intercept, both calculated using (x1,y1) and (x2,y2), and in which x and y are the longitude and latitude of GPS locations of the white storks in a particular day.

**Appendix S2**

**Definition of migratory days and stopover days**

To distinguish between migratory and stopover days, we used receiver operating characteristics (ROC) curves as a binary classifier for the daily latitudinal displacement. We assumed birds displace on a southerly direction on migratory days and then determined a daily displacement threshold to classify if birds were migrating or on a stopover. This threshold was computed as the optimal value maximizing sensitivity and specificity, using Youden’s J statistic. ROC curves, threshold and the Area Under the Curve (AUC) were computed using functions “roc” and “coords” and “auc” from package “pROC” (Robin *et al.* 2011), respectively. The ROC optimal threshold was 36.9km (which was then rounded to 37km), with specificity = 0.84, sensitivity = 0.64, and AUC = 0.769. The 37km threshold was then confirmed by visually inspecting the GPS tracks.

**References**

Robin, X., Turck, N., Hainard, A., Tiberti, N., Lisacek, F., Sanchez, J.C. & Müller, M. (2011). pROC: an open-source package for R and S+ to analyze and compare ROC curves. BMC Bioinformatics 12. DOI: 10.1186/1471-2105-12-77

**Appendix S3**

**Consistency in the timing and destination of autumn migration of adult white storks**

We analysed the individual consistency in the timing and destination of autumn migration of 5 adult white storks that were tracked for 2 (n=3) or 3 consecutive years (n=2). To do so, we first calculated the start and end of autumn migration using the spatio-temporal displacement method described in Soriano-Redondo *et al*. (2020) and extracted the longitude of the location where stork’s finished autumn migration. We then calculated the repeatability of the dates of start of autumn migration (in julian days) and using the “rpt” function of the R package “rptR” (Stoffel *et al.* 2017), which indicated that individuals were consistent in the timing of start of autumn migration (R = 0.79, SE = 0.22, *p-value* = 0.004). Using the same protocol, we calculated the repeatability of the longitudes of the location where individuals ended the autumn migration, which showed that white storks were highly consistent in the destination of autumn migration (R = 0.99, SE = 0.04, *p-value* < 0.001).

**References**

Soriano-Redondo, A., Acácio, M., Franco, A.M.A., Martins, B.H., Moreira, F., Rogerson, K. & Catry, I. (2020). Testing alternative methods for estimation of bird migration phenology from GPS tracking data. Ibis 162, 581-588, DOI: 10.1111/ibi.12809.

Stoffel, M. A., Nakagawa, S., & Schielzeth, H. (2017). rptR: repeatability estimation and variance decomposition by generalized linear mixed-effects models. Methods in Ecology and Evolution 8, 1639-1644, DOI: 10.1111/2041-210X.12797.
